# Antidepressant Effect of* Tetragonia tetragonoides (Pall.) Kuntze* Extract on Serotonin Turnover

**DOI:** 10.1155/2019/7312842

**Published:** 2019-02-03

**Authors:** Hyun Yang, Hye Jin Kim, Eui-Ju Hong, Bo-Jeong Pyun, Byung-Seob Ko, Hye Won Lee

**Affiliations:** ^1^Herbal Medicine Research Division, Korea Institute of Oriental Medicine (KIOM), Daejeon 34054, Republic of Korea; ^2^College of Veterinary Medicine, Chungnam National University, Daejeon, Republic of Korea

## Abstract

*Tetragonia tetragonoides (Pall.) Kuntze* (TTK) is a groundcover found along coastal areas of the Korean peninsula. TTK is traditionally used to improve women's health and treat gastrointestinal diseases. Use of herbal medicines in the treatment of mood disorders has recently been suggested as an alternative therapeutic strategy. In the present study, we determined that consumption of TTK extract ameliorated progression of depressive-like symptoms in ovariectomized (OVX) rats and further examined the mechanisms involved, i.e., synthesis, release, and reuptake(s) of serotonin (also known as 5-HT). We assessed the mRNA expression levels of tryptophan hydroxylases (TPH-1 and TPH-2) and serotonin transporter (SERT) as well as the reuptake activity of serotonin in RBL-2H3 cells. We also determined whether or not TTK extract regulates the serum level of serotonin and improves depressive-like symptoms in 0.5, 1, and 2% TTK-fed OVX female rats in a forced swimming test. Our results show that the mRNA levels of TPH-1 and SERT were significantly reduced, whereas the mRNA level of TPH-2 was dose-dependently elevated by TTK (50 and 100 *μ*g/mL) in RBL-2H3 cells. TTK significantly inhibited LPS- (lipopolysaccharide-) induced serotonin uptake in RBL-2H3 cells in a dose-dependent manner. The serum level(s) of serotonin was elevated by 1% and 2% TTK treatment in OVX female rats. Moreover, immobility time in the forced swimming test was reduced by 1% and 2% TTK treatment but not altered by 0.5% TTK treatment in OVX female rats. Taken together, these results indicate that TTK may significantly inhibit depressive-like symptoms due to upregulation of serotonin level(s) and regulation of serotonin reuptake activity. Thus, TTK may exert beneficial effects on depression during pre- or/and postmenopausal periods* via* modulation of serotonin synthesis and metabolism.

## 1. Introduction

Depression is a psychiatric disease as well as a chronic, recurring, and potentially life-threatening illness. The main symptoms of depression are characterized by a mood imbalance, loss of interest, and unhappiness [1]. The symptoms of depression during menopause are similar to general depression, although menopausal depression is highly affected by hormone fluctuation [2, 3]. Hormonal fluctuations such as elevation of follicle-stimulating hormone and reduction of ovarian hormone levels, i.e., estrogen and progesterone, are a common phenomenon during menopause [4].

*Tetragonia tetragonoides (Pall.) Kuntze* (TTK), known in Korea as Beonhaengcho, has similar textures and flavor properties as spinach. TTK therapy has been shown to alleviate menopausal symptoms and treat hepatic cell metabolism [5–7]. In a recent report, various herbal extracts improved menopausal symptoms such as hot flashes, weight gain, and involutional depression [8–10].* Hypericum perforatum*,* Rhodiola rosea*, and* Crocus sativus* have also been used as alternative therapeutics for the treatment of general depressive symptoms, inhibition of monoamine reuptake, and sensitization of neurotransmitter receptors [11–13].

Serotonin or 5-hydroxytryptamine (5-HT), a monoamine neurotransmitter, plays a critical role in the pathophysiology of mood disorders, i.e., anxiety disorder and depression [14, 15]. Serotonin controls central nervous system (CNS) function, including sleep, endocrine secretion, motor function, and cognition [16, 17]. Many antidepressant drugs target the release of serotonin and its transport system, including serotonin transporter (SERT) and inhibition of serotonin reuptake activity [18]. Selective serotonin reuptake inhibitor (SSRI) is used for treatment of depression, bone loss, and peripheral errors in postmenopausal women [19, 20]. The serotonergic systems and reproductive endocrinology have been linked in various reports, which are markedly related in mood change and behavior patterns [21, 22]. Fluoxetine is the only SSRI registered for the treatment of depression in postmenopausal women in the Unites States [23]. In addition, molecular imaging studies have observed reduction of brain SERT binding in major depressive disorders. Translational level of SERT might affect SSRI efficacy either directly or through adaptive changes in serotonergic function [24, 25].

Tryptophan hydroxylase (TPH) belongs to the enzyme superfamily of aromatic amino acid hydroxylases and is the regulator of serotonin synthesis and serotonin activity in the brain [26, 27]. There are two isoforms of TPH (TPH-1 and TPH-2), which mediate the synthesis of most peripheral serotonin and are predominantly expressed in the gut, thymus, spleen, and pineal gland [28, 29]. Especially, TPH-2 is a neuronal-specific enzyme that is predominantly expressed in the neurons of raphe nuclei in the brain stem, and it is the rate-limiting enzyme in serotonin synthesis as well as a key factor for serotonin transmission in the CNS [30, 31]. Recent studies have assessed the recurrence of patients suffering from depression, and targeting of TPH-2 was shown to improve the effectiveness of antidepressant medications [32, 33].

As mentioned before, we hypothesized that TTK might be effective in improving menopausal depression, which is known to have various pharmacological effects on improving the symptoms of menopausal symptoms, antiobesity, and hepatocyte apoptosis induction. In the present study, we examined the therapeutic effect of TTK on serotonergic system through the expression of TPHs, SERT and 5-HT reuptake activity in* in vitro *model. We also used ovariectomized (OVX) rats to investigate whether or not TTK can elevate serum levels of serotonin and improve immobility time in an* in vivo* model.

## 2. Materials and Methods

### 2.1. Preparation of TTK Extract

TTK was purchased from a commercial vender at Kwangmyung-Dang (Ulsan, South Korea). All raw materials and extracts were deposited at the Korea Institute of Oriental Medicine (KIOM 130081-3). Dried TTK (4 kg) was and extracted with 40 L of 70% ethanol for 3 days at 25-30°C. The 70% ethanol extracts were concentrated using a rotary evaporator and finally lyophilized in a freeze-dryer. Yield of TTK crude extract was 992.6 g (22.43% w/w).

### 2.2. Cell Lines and Cell Culture

The rat basophilic leukemia cell line RBL-2H3 was purchased from the ATCC (American Type Culture Collection). RBL-2H3 cells were maintained in Minimum Essential Medium (MEM) *α* containing 10% fetal bovine serum, 100 U/mL of penicillin, and 100 mg/mL of streptomycin in a humidified atmosphere containing 5% CO_2_ at 37°C.

### 2.3. Reagents and Antibodies

ASP (4-Di-1-ASP (4-(4-(Dimethylamino)styryl)-N-Methylpyridinium Iodide)) was obtained from Invitrogen (Carlsbad, CA, USA). LPS (Lipopolysaccharide) and Fluoxetine were purchased from Sigma-Aldrich (St Louis, MO, USA). Stock solutions were prepared in dimethyl sulfoxide (DMSO) and stored at -20°C. ASP and LPS were diluted in fresh medium before each experiment, and the final concentration of DMSO was <0.1%.

### 2.4. Reverse Transcription and Real-Time PCR

Total RNA was extracted using RNeasy Mini Kit (Qiagen, Hilden, Germany) following the manufacturer's instructions. cDNA was synthesized using 1 *μ*g of total RNA with a Thermo Scientific RevertAid First Strand cDNA Synthesis Kit (Thermo, Massachusetts, USA) and amplified by RT-PCR using AmpliTaq Gold DNA polymerase and Quantitative real-time PCR. cDNA was amplified using Premix ExTaq (TaKaRa Bio Inc., Shiga, Japan) with SYBR Premix EX Taq (TaKaRa Bio Inc.) using the BI PRISM 7500HT Sequence Detection System (Applied Biosystems, USA). Primer was synthesized by Macrogen Inc. (Seoul, South Korea). Actin expression was used as a control, and the primers used for RT-PCR are listed in Table 1.

### 2.5. Cell Growth Inhibition Assay

Cell viability was assessed using MTT assays. A total of 1x10^3^ cells/mL were seeded in 96-well plates, incubated for 16 h, and treated for 72 h with TTK at 37°C. After treatment, medium was replaced with an equal volume of fresh medium containing 2 mg/mL of 3-[4,5-dimethylthiazol-2-yl]-2,5-diphenyl-tetrazolium bromide (MTT, Sigma-Aldrich, St Louis, MO, USA) diluted in PBS and incubated for 4 h at 37°C, after which the medium was discarded. After confirming formation of formazan, medium was completely removed and analyzed by adding 150 *μ*L of DMSO to melt the formazan. Cell viability was determined by measuring absorbance at 540 nm using an ELISA microplate reader (Synergy HTX Multi- Mode Reader; BioTeK, Winooski, VT, USA).

### 2.6. 5-HT Uptake Assay

5-HT transport activity into RBL-2H3 cells was assessed by ASP assay. Briefly, RBL-2H3 cells were harvested at approximately 60-80% confluence and seeded in 24-well plates at a density of 5x10^5^ cells/well in 500 *μ*L of nucleoside-free MEM *α* containing 10% fetal bovine serum. Cells were untreated or treated with TTK at different concentrations (50 or 100 *μ*g/mL). After 16 h, medium was replaced with serum-free medium, followed by incubation for 4 h at 37°C. Cells were then washed with PBS buffer three times, incubated with medium containing 1 mM ASP, and dissolved in DMSO at 37°C for 1 h in the dark. Next, ASP-containing medium was removed, and cells were washed three times with PBS. Fluorescence density was measured using a multifluorescent microplate reader (Spectra-Max Paradigm Multi-Mode Microplate Reader; Molecular devices, Sunnyvale, California, USA) at *λ*ex=475 nm and *λ*em=605 nm.

### 2.7. Experimental Animals and Treatments

Female Sprague-Dawley rats (6-weeks-old, weight 130-150 g, total n=32, n=8 per group) were purchased from Orient Bio Inc. (Seongnam, South Korea) and allowed to adapt to laboratory conditions (temperature: 20 ± 2°C, relative humidity: 45 ± 5%, light/dark cycle: 12 h) for 1 week. Rats were anesthetized with an intraperitoneal (i.p.) injection of a Zoletil-Rompun-saline mixture (2:1:2). The dorsal midline of rats was incised, and the fallopian tube ligated. The ovaries were dissected using a surgical scissor, and the skin incision was sutured by surgical silk (6-0). The ovariectomized rats were fed AIN-76A-modified diet (37 En% carbohydrate, 20 En% protein, and 43 En% fat) as a vehicle control and supplemented with 0.5% (TTK0.5; w/w), 1% (TTK1.0; w/w), and 2% (TTK2.0; w/w) TTK for 8 weeks (from 8 to 16 weeks of age). Body weight (BW) and daily food intake were measured prior to and following the experimental feeding period. All animal experimental procedures were approved by the Ethics Committee of Veterinary Medicine of Chungnam University (Daejeon, South Korea).

### 2.8. Serum Serotonin Analysis

Blood samples were collected directly from the inferior vena cava using a 1-mL syringe at the end of the experiment. Serum was obtained by centrifugation at 4,000 x g for 10 min and stored at -70°C until use. Serum serotonin levels were measured using a Serotonin ELISA kit (Abnova; Taipei, Taiwan) according to the manufacturer's instructions.

### 2.9. Forced Swimming Test

Rats were subjected to a forced swimming test, and this method involves the rats becoming passive and immobile after a period of vigorous activity. The test apparatus was a polycarbonate cylinder (diameter: 300 mm, depth: 400 mm) filled with room temperature water. The rats were exposed to a pretest for 10 min prior to the experiment. Immobility was defined as no additional activity other than that necessary to keep the rat's head above the water.

### 2.10. Statistical Analysis

Data is presented as means ± SD. Paired Student's t-tests were used to compare each group or ANOVA with Tukey for multiple comparison tests using PRISM software (v6.0; Graph Pad, CA, USA).* P* values < 0.05 were considered to be statistically significant.

## 3. Results

### 3.1. Cytotoxicity Effect of TTK on RBL-2H3

**Figure 1:**
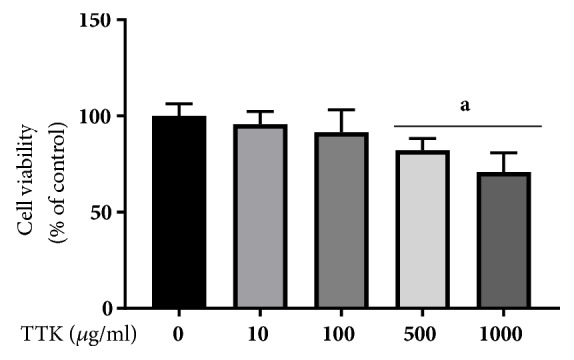
Cytotoxicity effect of TTK on RBL-2H3 cells. RBL-2H3 cells were treated with TTK for 72 hours, after which the cytotoxicity was measured by MTT assay. The percent of cell viability was calculated related to the untreated control as represented in 100%. Experiments were carried out in triplicate and data expressed as means ± SD. ^*a*^*p<*0.05 versus Control.

RBL-2H3 cells were treated with various concentrations of TTK for 72 h. Upon treatment with 10-1000 *μ*g/mL of TTK, viability rate of cells treated with 500 *μ*g/mL of TTK was approximately 80% compared to that of nontreatment. Based on the data, a subsequent experiment was conducted using a concentration of TTK below 500 *μ*g/mL (Figure 1).

### 3.2. Effects of TTK on TPH-1, TPH-2, and SERT mRNA Expression

**Figure 2:**
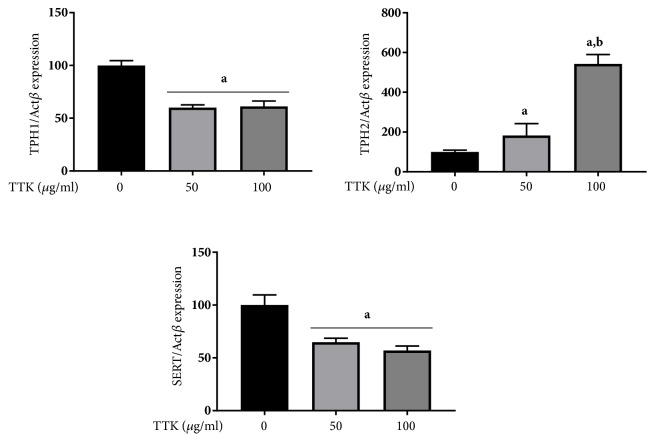
Effect of TTK on the mRNA expression of serotonin synthesis enzymes and serotonin transporter. Comparison of serotonin related mRNA expression levels between control condition (nontreated) or treated with TTK condition for 48 h. TPH-2, TPH-1, and SERT mRNA expressions were tested using RT-PCR in RBL-2H3 cell line. Experiments were carried out in triplicate and data expressed as means ± SD. ^*a*^*p<*0.05 versus Control; ^*b*^*p<*0.05 versus TTK 50 *μ*g/mL.

The effects of TTK on mRNA expression levels of TPH-1, TPH-2, and SERT, which are associated with serotonin synthesis and uptake, were assessed. Especially, TPH-2 is expressed in peripheral tissues of the brain, which is important in the regulation of mood disorders, whereas TPH-1 is not significantly expressed in the brain [34]. We demonstrated that treatment of RBL-2H3 cells with 50 and 100 *μ*g/mL of TTK significantly increased TPH-2 mRNA expression. On the contrary, TPH-1 and SERT expression decreased upon TTK treatment (Figure 2).

### 3.3. Effects of TTK on 5-HT Uptake by RBL-2H3 Cells

**Figure 3:**
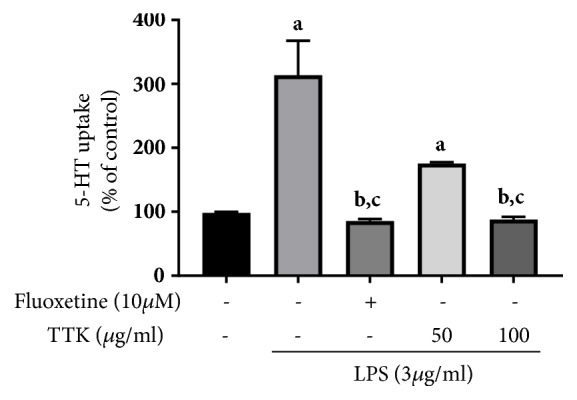
TTK inhibited LPS-induced 5-HT uptake in RBL-2H3. RBL-2H3 cells were seeded in a 24-well (5x10^5^ cell/well) and treated with 3 *μ*g/mL LPS, following treatment or absence of Fluoxetine (10 *μ*M) and TTK at different concentrations (50 *μ*g/mL; 100 *μ*g/mL) for 16 h. Experiments were carried out in triplicate and data expressed as means ± SD. ^*a*^*p<*0.05 versus Control, ^*b*^*p<*0.05 versus LPS 3 *μ*g/mL, and ^*c*^*p<*0.05 versus LPS 3 *μ*g/mL+TTK 50 *μ*g/mL.

To further explore the effects of TTK on 5-HT uptake by RBL-2H3 cells, 5-HT uptake was analyzed by measuring SERT activities based on ASP fluorescent intensities in RBL-2H3 cells. LPS is known to enhance 5-HT uptake by stimulating SERT, which plays a critical role in depression [34], whereas Fluoxetine is known to reduce 5-HT uptake by acting as an SSRI. The results show that 5-HT uptake significantly decreased upon TTK treatment in a dose-dependent manner similar to the effect of 10 *μ*M Fluoxetine (Figure 3).

### 3.4. Serum Serotonin Level and Immobility Time

**Figure 4:**
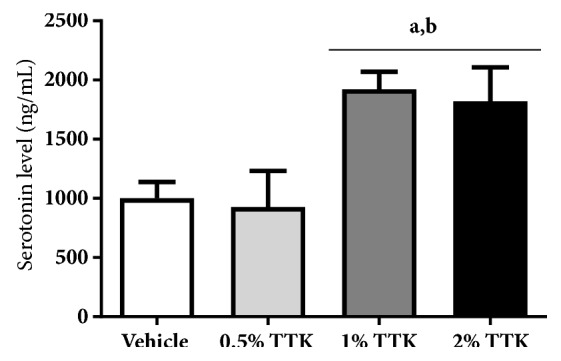
Serotonin structure and effect of TTK on serum serotonin level. Ovariectomized 8-wks-old female SD rats were fed AIN-76A modified diet (supplemented with 0.5%, 1%, and 2% TTK extract) for 8 weeks. The serum serotonin level was measured using a competitive enzyme-linked immunosorbent assay (ELISA) kit. All values represent means ± SD and ^*a*^*p<*0.05 versus vehicle group; ^*b*^*p<*0.05 versus 0.5% TTK group.

**Figure 5:**
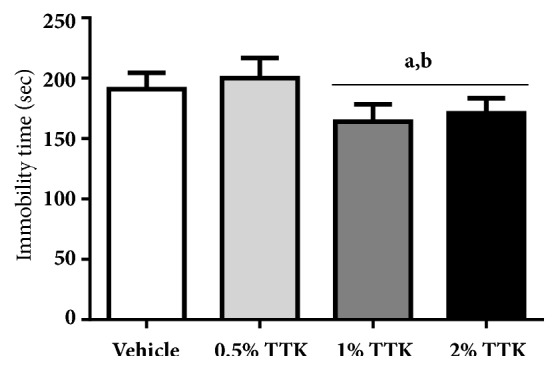
TTK inhibited immobility time in forced swimming test (FST). Ovariectomized rats (8-wks-old) were fed AIN-76A modified diet (supplemented with 0.5%, 1%, and 2% TTK extract) for 8 weeks. At 5 days before sacrifice, the mean of immobility time was measured as time spent floating by the rat without any actions in water. All values represent means ± SD and ^*a*^*p<*0.05 versus vehicle group; ^*b*^*p<*0.05 versus 0.5% TTK group.

Dietary supplementation with TTK extract affected serum levels of serotonin in rats. As shown in Figure 4, serum serotonin levels were significantly elevated by supplementation with 1% or 2% TTK extract compared to the vehicle group. However, serum levels of serotonin were not altered in the 0.5% TTK-treated group. The effect of TTK on immobility time in rats is shown in Figure 5. The doses of TTK extract (1% and 2%) significantly reduced the duration of immobility in comparison with vehicle control, but its effect was not shown in 0.5% TTK-treated group. The maximal antidepressant effects of TTK extract were obtained with 1% TTK diet, and there were no significant differences between 1% and 2% TTK. These results indicate that TTK extract improved immobility in the forced swimming test, suggesting this beneficial compound ameliorated depression in rats.

## 4. Discussion

Herbal extracts have recently been reported as complementary therapies for antidepressant purposes, and this type of treatment is expected to prevent onset of mood disorders and regulate serotonin reuptake activity [35, 36]. However, these alternative remedies are limited in the treatment of severe depression [9, 37]. In the present study, we examined TPH-1 and TPH-2 mRNA expression levels in RBL-2H3 cells after treatment with 0, 50, and 100 *μ*g/mL of TTK. Quantitative real-time RT-PCR analysis revealed that mRNA expression of TPH-2 was dose-dependently up-regulated by TTK extract (50 and 100 *μ*g/mL). In addition, mRNA expression of TPH-1 was significant reduced following treatment with all concentrations of TTK extract. TPH is an isoenzyme, and it is involved in the serotonin synthesis and has two different isoforms, TPH-1 and TPH-2 [33, 38]. TPH-1 is utilized mainly in peripherals such as enterochromaffin cells of the gut, whereas TPH-2 is the predominant gene transcribed in the brain [33, 38]. Interestingly, the serotonins of brain and peripherals might be linked, acting as a regulatory interface for neurotransmitters [39]. Up-regulation of TPH-2 transcription induces TPH activity and 5-HT release, which influence synaptic 5-HT activity [40, 41]. We also observed that SERT transcription was significantly down-regulated by TTK extract in RBL-2H3 cells.

SERT is a member of neurotransmitter-sodium symporter, and it plays an important role in the released and extinguished serotonin through transport across the presynaptic membrane [41], and numerous studies have reported the antidepressant effects of reduction of SERT mRNA expression [42, 43]. In the current study, we investigated whether or not reduction of SERT transcription can regulate 5-HT reuptake activity in TTK-treated RBL-2H3 cells. Our results show that 5-HT reuptake activity was dose-dependently reduced by TTK treatment in LPS-induced RBL-2H3 cells, and this pattern was also observed in the SSRI (Fluoxetine; 10 *μ*M)-treated group. The serotonergic system has long been related in the pathogenesis of depression, and the potential evidence involves the inhibition of depression by SSRIs [44]. Other studies have indicated that herbal extracts and their ingredients may exert SSRI-like effects in* in vitro* and* in vivo* models [44, 45]. These data suggest that TTK extract may be critical for expression of TPH-1, TPH-2, and SERT as well as inhibition of 5-HT reuptake activity in RBL-2H3 cells via a mechanism similar to those of SSRIs.

In the present study, we determined that rats fed TTK extract (0.5%, 1%, and 2%) for 8 weeks showed up-regulation of serotonin levels in serum or the reduction of immobility in behavioral test upon 1% and 2% TTK treatment, and the data have already been collected by patent office in South Korea [46]. Peripheral serotonin has long been reported to be the suitable marker for diagnosis of depression, as blood serotonin shares a similar serotonin uptake and release mechanism as serotonergic neurons [47–49]. Furthermore, the forced swimming test is widely used to screen potential antidepressive effects* in vivo*, and antidepressants reduce immobility time in this test [50, 51]. Several important clinical implications have emerged based on the finding that 5-HT is associated with the behavioral effects of SSRIs in the forced swimming test [50, 51].

The present study assessed the expression of TPH-1, TPH-2, and SERT as well as 5-HT reuptake activity in RBL-2H3 cells and described serum levels of serotonin and immobility time in OVX female rats. Based on the findings, it appears that TTK may be a potent therapeutic agent for the treatment of depression based on its regulation of serotonin-mediated genes and peripheral serotonin level(s) in our* in vitro* and* in vivo* model. However, TTK crude extract contains variety of compounds, and further studies are needed on fractions or partial purification of the extract to identify the active pharmaceutical ingredient among the TTK-derivative compounds.

## Figures and Tables

**Table 1:** Sequence identification and primers used for RT-PCR analysis.

No.	Gene	Forward Primer (5′ to 3′)	Reverse Primer (5′ to 3′)	Annealing Temperature (°C)	Product Size (bp)
1	TPH-1	ACCATCTTC CGAGAGCTGAA	GATGGAAAACCCTGTGCGTT	58°C	162
2	TPH-2	ATCCCAAGTTTGCTCAGTTTT	GATGGACGAAAGTAACCCTG	58°C	167
3	SERT	AACTGGCAGAAACTCTTGGA	GAAGATGACGAA GCCAGAGA	58°C	195
4	Actin	TACGTCGCCCTGGATTTT	ATGAAAGAGGGCTGGAAGAG	60°C	149

## Data Availability

All data used to support the findings of this study are included within the article.
